# Outpatient Percutaneous Release of Trigger Finger: A Cost Effective and Safe Procedure

**DOI:** 10.5704/MOJ.1703.021

**Published:** 2017-03

**Authors:** Z Marij, Q Aurangzeb, HR Rizwan, R Haroon, MH Pervaiz

**Affiliations:** Department of Orthopaedics, Aga Khan University Hospital, Karachi, Pakistan

**Keywords:** Trigger finger, percutaneous release, outpatient treatment, cost-benefit analysis

## Abstract

**Introduction:**

Trigger finger is a common cause of pain and disability of the hand. Percutaneous release results in earlier functional recovery and patient satisfaction. This is a rapid and cost-effective method which saves a surgical procedure and results in better functional outcome.

**Materials and Methods:**

This is a prospective observational study conducted on fifty-two fingers and thumbs in 52 patients treated from 1st July 2014 till 31st December 2014, in the Orthopaedic Section, Department of Surgery, Aga Khan University Hospital, Karachi, Pakistan. All the baseline characteristics of the patients, like demographics, symptoms, Quinell's criteria and functional outcome were recorded. The patients were treated at our hospital with trigger finger, managed with percutaneous release using an 18 gauge needle and followed up for a minimum period of three months. The follow-up information included range of motion scoring, patient satisfaction and overall outcome of the procedure in terms of patient acceptance. The data was analyzed to determine the functional outcome at three months.

**Results:**

There was complete release of A1 pulleys in 52 out of 52 digits (100%) in the patients undergoing percutaneous release and significant patient satisfaction. No recurrence was observed.

**Conclusion:**

Percutaneous release of trigger finger with needle was not only associated with excellent functional outcome and recovery in terms of patient satisfaction and range of finger motion three months post-procedure but also was found to be cost effective.

## Introduction

Trigger finger is one of the common causes of pain and disability of the hand^1^,^2^. This condition results in painful catching^3^ or popping of the involved flexor tendon^4^ as the patient flexes and extends the digit. On occasions, the digit will lock in flexion and require passive manipulation of the digit for full extension. Over a period of time, guarding and reluctance on the part of the patient to fully move the digit can lead to secondary contractures^5^ at the proximal interphalangeal joint. The phenomenon of tendon entrapment is due to mechanical impingement of the digital flexor tendons as they pass through a narrowed A1 pulley^6^ at the level of the metacarpal head.

The condition has a reported annual incidence of 28 cases per 100 000 population^7^, or a lifetime risk of 2.6% in the general population^7^. This rises to 10% in patients with diabetes^8^. Secondary trigger finger can be seen in patients with diabetes^9^, gout, renal disease, rheumatoid arthritis^10^ and other rheumatic diseases and is associated with a worse prognosis after conservative or surgical management^1^. The most common form is the primary type^4^, found in otherwise healthy middleaged women with a frequency two to six times that seen in men^11^. The patients are classified from grade I which is pretriggering to grade IV with flexion contracture. In patients with multiple trigger digits, the most commonly affected is the thumb^12^, followed by the ring, middle, little, and index fingers^3^. Two peaks in incidence occur the first under the age of eight and the second (more common) in the fifth and sixth decades of life^1^. This bimodal distribution represents two different clinical groups; not only for age but also in incidence, sex distribution, digit affected, treatment, and outcome^1^.

Treatment comprises of local corticosteroid injections^13^, splintage^14^, hydrotherapy, analgesics^11^, percutaneous release and eventual open surgery in patients not responding to the above regimens. Percutaneous release^15^ results in earlier functional recovery and patient satisfaction. This is a rapid and cost-effective method^16^, ^17^, which saves a surgical procedure and results in better functional outcome. In the current study we performed percutaneous release of trigger finger with 18 gauge needle, followed the patients for at least three months and recorded their outcomes in terms of patient satisfaction and range of motion.

## Materials and Methods

The current study is a prospective observational study conducted at Aga Khan University Hospital, Karachi, Pakistan for duration of six months from 1st July, 2014 to December, 2014. A total of 52 patients were included in the study, the inclusion criteria being all adult patients (age>18years) presenting with trigger finger diagnosed on the basis of clinical symptoms like pain, catching and stiffness while those patients experiencing recurrence of the same digit and those on anticoagulants were excluded. Data was collected using a structured proforma. Patients were recruited on presentation to the orthopaedic consulting clinics according to the selection criteria. The purpose, procedure, risks and benefits of the study were explained to the patients and a formal written consent was taken. Patients were followed up for at least three months after the procedure and on final follow-up patients underwent postprocedure assessment of finger range of motion using a goniometer measuring all the three ranges (1. <0°, 2. 0>5°, 3. 5°-10°). Patient satisfaction with the procedure was assessed through direct questioning and a satisfactory or very satisfactory response was considered acceptable in the final follow-up. Data was analyzed via SPSS v20. Results were presented as mean for continuous variables of age and as frequency/percentage for gender, hand and finger involved, finger range of motion and patient satisfaction.

**Fig. 1:**
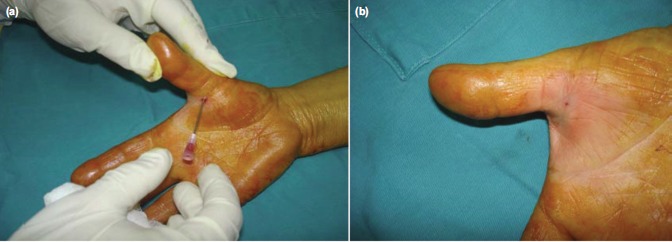
(a) Insertion of 18 gauge needle to release A1 pulley and (b) photograph after completion of the procedure.

All patients underwent percutaneous release with 18 gauge needle in the consulting clinic after a formal written consent and by a single orthopaedic surgeon with a certified hand fellowship. Patients were positioned sitting on a chair to the right of the operating hand surgeon with easy access to the finger involved. No antibiotics were given prophylactically. The procedure was done under local anesthesia. The local anesthetic comprised of a 2% solution of Lidocaine with adrenaline^18^, ^19^, infiltrated with a long 25 gauge needle over the volar surface of the distal palmar crease of the affected digit. Then, using an 18 gauge needle, the A1-pulley over the metacarpo-phalangeal joint was released in a proximal to distal stroking motion with the sharp edge of the needle, usually requiring one to two sweeps with resultant release of the A1-pulley. This resulted in an immediate relief of symptoms of pain and catching. No suture was applied and a single saniplast was applied over the wound. (Fig. 1)

In the post-procedure period all patients were asked to move their fingers actively as required. They were followed up in clinic after one week and then at three months postprocedure to assess functional range of motion.

## Results

A total of 52 adult patients with trigger fingers were included in this study. Mean age was 49.65 years with a range of 19-69 years. The most frequent involved digit was thumb (38.5%) followed by index, middle and ring fingers with 28.8%, 25% and 7.7% respectively. The most frequent presenting symptom was pain (48.1%) followed by stiffness and catching with 28.8% each. (Table I). There was complete relief of symptoms (pain/locking/catching) in 52 out of 52 fingers (100%). No patient had any recurrence in the three months period (Table II). Correlation of hand and grading of trigger finger was also analyzed (Table III). Subjective and objective outcomes after three months were recorded (Table IV).

**Table I:** Symptoms, grading and degree of hyperextension

Clinical features	Number (Percentage)
Symptoms at presentation	
Catching	12 (23.1%)
Pain	25 (48.1%)
Stiffness	15 (28.8%)
Trigger finger grading	
Grade I- Pain and nodularity	12 (23.1%)
Grade II- Self correctable triggering	20 (38.5%)
Grade III- Manually correctable triggering	20 (38.5%)
PIP Joint hyperextension (in degrees)	
0-5	22 (42.3%)
5-20	30 (57.7%)

**Table II:** Information of patients

Patient Characteristic	Types	Number (Percentage)
Mean age (years)		49.65+/-13.14 SD
Gender	Male/Female	23/29 (44.2%/55.8%)
Hand involved	Right/Left	25/27 (48.1%/51.9%)
Hand dominance	Right/Left	42/10 (80.8%/19.2%)
Digit involved	Thumb	20 (38.5%)
	Index	15 (28.8%)
	Middle	13 (25.0%)
	Ring	04 (7.7%)

**Table III:** Hand affected and trigger finger grading

Hand affected	Trigger finger grading (Quinell’s Criteria)				
	Pain and nodularity	Triggering, self-correctable	Triggering, manually correctable	Irreducible	Total
Right	5	12	8	0	25
Left	7	8	12	0	27
Total	12	20	20	0	52

**Table IV:** Outcomes

	Number (Percentage)
Objective outcome at 3 months	
Satisfactory	47 (90.4%)
Unsatisfactory	5 (9.6%)
Subjective outcomes at 3 months	
Unsatisfactory	6 (11.5%)
Satisfactory	22 (42.3%)
Very satisfactory	24 (46.2%)
PIP Joint Hyperextension (in degrees) at 3 month	
0-5	1 (1.92%)
5-10	51 (98.18%)

## Discussion

Currently open release remains the mainstay of the treatment for trigger fingers. Fingers are still managed by open surgical release in areas where there is limited expertise for percutaneous release. Conservative management is also practised in patients who do not want to undergo surgical release and includes corticosteroid injections. This results in unwarranted surgical procedures on one hand and prolonged conservative management on the other hand with persistent patient suffering in both instances.

The major disadvantage of open treatment is a small but definite incidence of complications directly related to surgical intervention like infections, pain, scar formation, joint stiffness or weakness, bowstringing of the flexor tendons due to pulley injuries and digital nerve or artery damage^18^.

The percutaneous surgical release technique performed by Eastwood *et al*^20^ is a convenient, minimally invasive, economical method with a very low complication rate, and is becoming more popular than open surgery. Mohsen^21^ in his study, reported 97% success rate of percutaneous release in 40 trigger digits, the thumb being the most common digit, similar to our study which showed 100% successful release and the thumb was also the most common digit involved.

Sahu *et al*^9^ reported successful results in 95.6% patients (excellent in 82.6% and good in 13%). In another study Ramy^22^ analyzed a study of 42 patients in which he reported incomplete release of A1 pulley in three fingers 6.97% and superficial flexor tendon laceration in six fingers (13.95%). Mishra *et al*^21^ reported a case series of percutaneous release of trigger fingers with the tip of 20 gauge hypodermic needle in which they reported success rates of 95.4%, with no recurrence and concluded that the procedure was safe and effective with lower complication rates compared to open surgery, comparable to our study. There is a close anatomical relationship between the radial digital neurovascular bundle of the thumb and the A1 pulley. Various studies recommend not to perform a percutaneous release of trigger thumb and proceed for open release. Pope and Wolfe^23^ performed percutaneous release in 25 cadaveric palms and found that the radial digital nerve was as close as within 2 to 3 mm of the needle site in three of five thumbs and five of five index fingers. Ferhat Guler *et al*^24^ reported digital nerve injury in 5.7% patients who underwent percutaneous release of trigger thumb. In our study, none of the patient had such injury.

Moreover there is a significant cost difference between the two procedures. Open release is dealt as a day-care procedure with multiple logistics such as operative room charges, drapes, sterile instruments and suture material and costs 51,200 PKR (Pakistani Rupees) in our hospital. Percutaneous release of trigger finger on the other hand is done in the clinic, just requires a local anesthesia, pair of sterile gloves, sterile sheet, and 18-gauge needle and only 7200 PKR is charged from the patient, almost seven times cheaper and cost-effective than the open technique.

The limitations of current study were small sample size and single arm study.

## Conclusion

This study showed that percutaneous technique for release of trigger finger is safe, cost effective technique with significant patient satisfaction. It is performed in the clinic, just requires an anesthetic and a disposable 18 gauge needle and has shown promising results while on other hand open release requires a day care procedure, use of sterilized equipment, skin incision and a suture. With a resource constraint country, percutaneous release of trigger finger proves to be a highly cost-effective method. The only pitfall of percutaneous technique is its blind nature but with very few complications. This study impels the reviewers and opens the grounds for further elaborated and extensive studies in future.

## Disclosure

No conflicts of interest were declared by the authors.
